# Onset Symptom Clusters in Multiple Sclerosis: Characteristics, Comorbidities, and Risk Factors

**DOI:** 10.3389/fneur.2021.693440

**Published:** 2021-07-06

**Authors:** Vladeta Ajdacic-Gross, Nina Steinemann, Gábor Horváth, Stephanie Rodgers, Marco Kaufmann, Yanhua Xu, Christian P. Kamm, Jürg Kesselring, Zina-Mary Manjaly, Chiara Zecca, Pasquale Calabrese, Milo A. Puhan, Viktor von Wyl

**Affiliations:** ^1^Swiss MS Registry, Epidemiology, Biostatistics and Prevention Institute, University of Zurich, Zurich, Switzerland; ^2^Department of Psychiatry, Psychotherapy and Psychosomatics, Psychiatric University Hospital Zurich, Zurich, Switzerland; ^3^Department of Neurology, Inselspital, University Hospital Bern and University of Bern, Bern, Switzerland; ^4^Neurocentre, Luzerner Kantonsspital, Lucerne, Switzerland; ^5^Department of Neurology and Neurorehabilitation, Rehabilitation Centre Kliniken Valens, Valens, Switzerland; ^6^Department of Neurology, Schulthess Clinic, Zurich, Switzerland; ^7^Department of Health Sciences and Technology, ETH Zurich, Zurich, Switzerland; ^8^Department of Neurology, Multiple Sclerosis Center (MSC), Neurocenter of Southern Switzerland, Lugano, Switzerland; ^9^Faculty of Biomedical Sciences, Università della Svizzera Italiana (USI), Lugano, Switzerland; ^10^Division of Molecular and Cognitive Neuroscience, University of Basel, Basel, Switzerland

**Keywords:** multiple sclerosis, onset symptoms, comorbidity, risk factors, latent class analysis, epidemiology

## Abstract

**Background:** Multiple sclerosis (MS) symptoms are expected to aggregate in specific patterns across different stages of the disease. Here, we studied the clustering of onset symptoms and examined their characteristics, comorbidity patterns and associations with potential risk factors.

**Methods:** Data stem from the Swiss Multiple Sclerosis Registry, a prospective study including 2,063 participants by November 2019. MS onset symptoms were clustered using latent class analysis (LCA). The latent classes were further examined using information on socio-demographic characteristics, MS-related features, potential risk factors, and comorbid diseases.

**Results:** The LCA model with six classes (frequencies ranging from 12 to 24%) was selected for further analyses. The latent classes comprised a multiple symptoms class with high probabilities across several symptoms, contrasting with two classes with solitary onset symptoms: vision problems and paresthesia. Two gait classes emerged between these extremes: the gait-balance class and the gait-paralysis class. The last class was the fatigue-weakness-class, also accompanied by depression symptoms, memory, and gastro-intestinal problems. There was a moderate variation by sex and by MS types. The multiple symptoms class yielded increased comorbidity with other autoimmune disorders. Similar to the fatigue-weakness class, the multiple symptoms class showed associations with angina, skin diseases, migraine, and lifetime prevalence of smoking. Mononucleosis was more frequently reported in the fatigue-weakness and the paresthesia class. Familial aggregation did not differ among the classes.

**Conclusions:** Clustering of MS onset symptoms provides new perspectives on the heterogeneity of MS. The clusters comprise different potential risk factors and comorbidities. They point toward different risk mechanisms.

## Introduction

Multiple sclerosis (MS) research has always faced the challenge of significant heterogeneity of phenotypes and variety of potential risk mechanisms. This applies to research both across and within the most common MS subtypes—the primary-progressive form (PPMS), the relapsing-remitting form (RRMS), and its secondary-progressive continuation, the SPMS. Heterogeneity became a salient topic in the 1990s on the basis of research on diverse pathogenic mechanisms in MS (1, 2). Earlier epidemiological investigations had already documented sex-specific changes in MS prevalence (3), a readily observable indicator of risk heterogeneity. In the early 1980s, Canadian immunologists identified two clearly distinguishable MS types based on occurrence of past infectious events before the MS onset (4). More recently, machine learning algorithms have emerged as promising tools for building classifications on multiple characteristics (5). Last but not least, disease-modifying treatments have provided additional corroborating evidence for the existence of heterogeneous types in MS by showing that immunomodulatory drugs have varying effects across patients (6).

In terms of methodology, appropriately assessing, and reproducing the heterogeneity of subtypes or—equivalently—heterogenous patient subgroups in complex diseases is crucial (7), and MS can in fact be regarded as a complex disease (8). This study is a another effort in this vein: it focuses on the onset symptoms of MS in order to identify subgroups of persons sharing the same symptom configurations at the beginning of the disease.

MS comprises a broad spectrum of symptoms, including vision impairment, sensorimotor deficits, paralysis, dizziness, balance problems, spasms and pain, paresthesia, bladder, and intestinal dysfunction, as well as neurobehavioral, neuropsychiatric, and various further problems. So far, only a few studies have examined whether MS symptoms aggregate into specific patterns (9–12). The focus of these studies was on the consequences and outcomes related to specific symptom clusters, independently of when the symptoms occurred during the disease course. Onset symptoms have largely escaped the attention of MS researchers who applied classification analyses.

Onset symptoms represent a potential link to processes that precede the clinical onset of MS. Some of these initial processes might emerge long before the first symptoms occur and include a variety of risk mechanisms, whereas other processes seem to occur closer to the clinical onset. Recent clues have come from research on the MS prodrome (13–15), which documented a more intense use of health services for mental and physical problems over several years before the clinical manifestation of MS. Research on the clinically isolated syndrome (CIS) (16) and the radiologically isolated syndrome (RIS) (17, 18) has further corroborated this focus.

In sum, focusing on early stages of MS may foster knowledge on processes preceding and succeeding the clinical onset. In this study, we clustered MS onset symptoms reported by participants from the Swiss MS Registry (SMSR). In order to characterize the clusters, we examined their socio-demographic features, MS-specific characteristics and associations with potential risk factors, with preceding infectious diseases and with comorbid inflammatory diseases.

## Materials and Methods

### Study and Participants

The SMSR started in June 2016 (19, 20) as a nationwide patient-centered longitudinal survey funded by the Swiss MS Society (see http://www.Clinical-Trials.gov identifier: NCT02980640). Participants in the SMSR are adults (≥18y) with a CIS or with an MS diagnosis, confirmed by their treating physician. A separate part of the SMSR is reserved for relatives and close friends. Participation of persons with MS (PwMS) is limited to those living in Switzerland or receiving care through the Swiss health system, and is based on informed consent.

All SMSR surveys were provided in the three national languages (German, French, or Italian) and were completed either through an online system or via paper-pencil versions. The participants entered the surveys by completing a short initial questionnaire followed by a comprehensive baseline questionnaire. Further questionnaires followed semi-annually and were confined to MS-specific subjects (diagnosis process (21, 22), patient satisfaction (23), profession and job, depression, and nutrition.

Up to November 2019, a total of *N* = 2,159 participants were enroled in the SMSR. Data for 2,063 participants had been checked and were available for the analysis. Information on onset symptoms and other basic information (socio-demographic data, clinical MS type, time of diagnosis, familial aggregation, MS-specific therapies) was taken from the short initial questionnaire. Data on potential risk factors and comorbid diseases/disorders stem from the baseline questionnaire. Information on MS type was also updated using data from subsequent questionnaires.

### Analyzed Variables

The onset symptoms covered in this analysis comprise vision, fatigue, speech, dysphagia, weakness, paralysis, paresthesia, dizziness, pain, gait, balance, bladder, spasms, tics, tremor, bowel, epilepsy, sexual problems, memory, and depression symptoms. For clustering analyses, the least frequent symptoms (<8% of the sample) were omitted. A sum variable was created to represent the symptom load.

The clinical MS type was defined by three categories, with CIS and relapsing-remitting MS in a composite RRMS category separate from the PPMS and the SPMS types. The age of onset was represented by two variables: either by the diagnosis date or based on first symptoms reported by the participant. In the latter case, missing age values and age values higher than those of diagnosis date were replaced by the age of recieving a diagnosis. In outliers (>3 standard deviations for the difference between the age of first symptoms and the age of diagnosis) the symptom-based onset variable was set to missing. The disability status was represented by an expanded disability status scale (EDSS) proxy, i.e., a three-category variable based on walking distances, use of walking aids and use of a wheelchair, that was proposed by our group in a former study [for more details see (24)]. Further MS-related variables comprised the number of relapses, use of immunomodulatory therapies (current and lifetime), and current use of alternative medicine. Familial aggregation was defined as having any first-degree relatives with MS.

Health-related quality of life was assessed by a visual analog scale, which was used as a supplement to the European Quality of Life 5-Dimension Scale (EQ5d) (25). In addition, a screening instrument for depression, the WHO-5 Well-Being Index (26) was applied.

Sociodemographic variables included sex, birth year and age, education level (high school vs. lower level), nationality (Swiss vs. other). Potential risk factors that were assessed from the beginning of the SMSR comprised smoking (here dichotomized as lifetime smoker vs. other), alcohol consumption (daily/weekly, less frequent, never), and body mass index (BMI). Among comorbid diseases and disorders, only the most frequent ones were introduced in the analysis:

- mononucleosis
- angina/tonsillitis
- skin diseases (acne, psoriasis)
- herpes/fever blisters
- cystitis
- migraine
- gastro-intestinal disorders (colitis ulcerosa, Crohn's disease, gastritis, irritable bowel syndrome)
- atopic diseases (hay fever, asthma, eczema, food allergies)
- drug allergy
- other autoimmune disorders.

In all comorbid diseases and disorders, this is lifetime prevalence data. No information about the onset year was available. However, most of the listed conditions typically emerge before the age of onset of MS.

### Statistical Analysis

The analysis design comprised four steps: descriptive statistics, clustering of onset symptoms, followed by the conventional design incorporating bivariate associations and multinomial regression analyses based on the symptom clusters. In the multinomial regression analysis, the variable representing the classes was regressed on a selection of potential risk factors and comorbidities that were significant or trend significant at the 5%-level in bivariate association analyses. The clinical MS type was not included since we considered it as an outcome rather than as a predictor of the latent classes. Backward and forward selection outcomes were compared in order to confirm the results. The inclusion of a predictor at each step was based on *p* < 0.05, its exclusion on *p* > 0.10.

MS onset symptoms were clustered using latent class analysis (LCA), which is a classification model like factor analysis or cluster analysis. In contrast to factor analysis, which is a variable-centered approach that places variables along dimensions or factors, LCA is a person-centered approach, i.e., it aims to group individuals into homogeneous classes (27, 28). In LCA, the proportion of participants in each class is determined by class probabilities. Depending on the selection of variables, the classes in the LCA can be interpreted as representing subtypes of a disease.

Initially, LCA models with one to seven latent classes were routinely fitted to the data in order to determine the optimal number of latent classes in the final model. We considered several fit indices: Akaike information criterion (AIC), the Bayesian information criterion (BIC), and the sample-size adjusted BIC (ABIC) as well as the Lo-Mendell-Rubin likelihood ratio test (LMR-LRT) (29). Typically, we prefered models with a number of classes between the number suggested by the BIC and the number suggested by the AIC (30). Model selection was furthermore determined by the distinction between the classes, their size and their theoretical adequacy.

In analyses that focus on pattern recognition, either through a classification model such as an LCA or, implicitly, in analyses of groups of markers and disorders, we explicitly refrain from performing adjustments for multiple testing [see also (31)]. The analyses were conducted with Mplus (version 7 for Macintosh) and SPSS (version 23.0 for Macintosh).

## Results

The analysis is based on 2,063 persons with MS or CIS, of whom 1,503 were women (72.9%) and 560 (27.1%) men. Most of the participants had RRMS/CIS [*n* = 1,403 (69.5%)], followed by SPMS [*n* = 393 (19.5%)] and by PPMS [222 (11.0%)] (*n* = 39 missings).

The onset symptoms are listed in Table 1. The most frequent symptoms (proportions >25%) were paresthesia, vision problems, fatigue, weakness, gait problems, balance problems, and paralysis. The LCA of onset symptoms (*n* = 1,942, 115 data points missing due to lacking information or to symptoms not included in the LCA) yielded a preferable solution with six classes. The choice was based on the BIC values (lowest value), the decelerated decline of the AIC and ABIC values and the interpretability of the latent classes (see model fits of 1–7 classes in Supplementary Table 1). To facilitate comparisons, the outcomes of the five and the seven class solution are also described below.

**Table 1:** Frequencies of MS onset symptoms (%) in the Swiss Multiple Sclerosis Registry; overall, by sex and by MS type; *p*-values after χ^2^-test.

		**Sex**			**MS type**			
	**All**	**Men**	**Women**	***P*-value**	**PP**	**RR**	**SP**	***P*-value**
Balance problems	575 (28.6)	160 (28.8)	415 (28.2)	0.564	71 (34.1)	387 (28.0)	106 (27.5)	0.166
Bladder problems	225 (11.1)	59 (10.8)	166 (11.2)	0.788	30 (14.0)	144 (10.4)	46 (11.8)	0.252
Bowel problems	185 (9.2)	37 (6.8)	148 (10.0)	0.026	25 (11.7)	125 (9.0)	35 (9.1)	0.442
Depression symptoms	247 (12.2)	61 (11.2)	186 (12.6)	0.406	28 (13.0)	171 (12.4)	43 (11.1)	0.730
Dizziness	423 (20.8)	102 (18.7)	321 (21.6)	0.156	30 (14.0)	319 (23.0)	66 (17.0)	0.001
Epilepsy	21 (1.0)	5 (0.9)	16 (1.1)	0.746	2 (0.9)	15 (1.1)	3 (0.8)	0.860
Fatigue	707 (35.2)	163 (29.4)	544 (37.1)	0.004	81 (38.9)	510 (37.0)	107 (27.9)	0.003
Gait problems	642 (31.7)	196 (35.9)	446 (30.2)	0.014	115 (53.7)	376 (27.1)	137 (35.5)	0.000
Memory problems	174 (8.6)	40 (7.3)	134 (9.0)	0.216	18 (8.4)	130 (9.4)	21 (5.4)	0.043
Pain	286 (14.1)	66 (12.0)	220 (14.9)	0.104	36 (16.7)	200 (14.4)	44 (11.3)	0.154
Paralysis	526 (26.0)	141 (25.8)	385 (26.0)	0.925	60 (27.9)	355 (25.6)	102 (26.5)	0.755
Paresthesia	1,194 (58.8)	307 (56.2)	887 (59.7)	0.155	94 (44.1)	851 (61.2)	226 (57.9)	0.000
Sexual problems	121 (6.0)	42 (7.7)	79 (5.3)	0.045	21 (9.8)	71 (5.1)	26 (6.7)	0.020
Spasms	188 (9.3)	55 (10.1)	133 (9.0)	0.444	42 (19.6)	106 (7.6)	34 (8.8)	0.000
Speech problems	137 (6.7)	40 (7.3)	97 (6.5)	0.545	9 (4.2)	105 (7.6)	19 (4.9)	0.050
Swallowing problems	53 (2.6)	16 (2.9)	37 (2.5)	0.594	8 (3.7)	34 (2.4)	10 (2.6)	0.581
Tics	87 (4.3)	26 (4.7)	61 (4.1)	0.518	11 (5.1)	56 (4.0)	19 (4.9)	0.646
Tremor	117 (5.7)	29 (5.3)	88 (5.9)	0.584	13 (5.9)	81 (5.8)	21 (5.4)	0.943
Vision problems	829 (40.8)	200 (36.6)	629 (42.3)	0.021	62 (28.6)	572 (41.1)	184 (47.5)	0.000
Weakness	638 (31.6)	159 (29.2)	479 (32.5)	0.164	78 (37.0)	427 (30.9)	122 (31.6)	0.208

**Figure 1:**
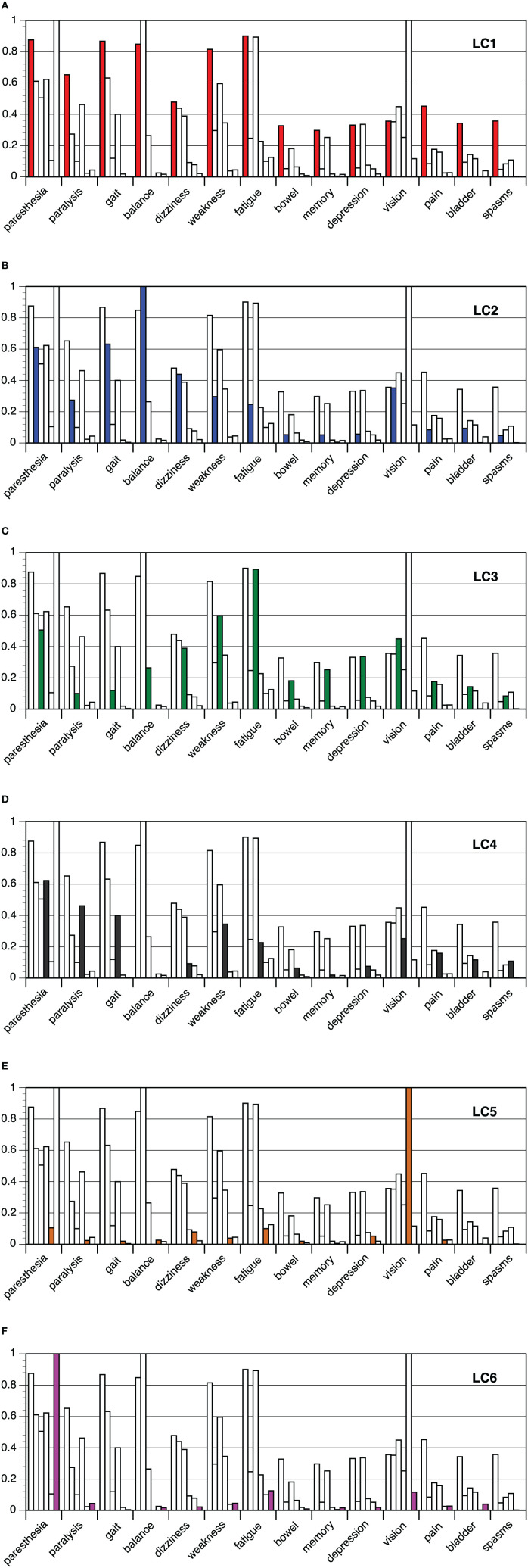
**(A–F)**: Latent class analysis of onset symptoms in multiple sclerosis: probabilities of onset symptoms per class 1–6.

The probabilities of the onset symptoms per latent class are shown in Figures 1A–F. Separate analyses for men and women are documented in Supplementary Tables 2, 3, Supplementary Figures 1, 2). The results were almost perfectly comparable with six classes in the larger subsample of women and five classes in the smaller subsample of men.

On one side of the spectrum the multiple symptoms class (LC1, 14.1%) was located, with high probabilities across most of the onset symptoms. On the other side of the spectrum, there were the classes with a solitary onset symptom: LC5 (vision problems, 21.3%) and LC6 (paresthesia, 15.4%). Between these poles three further classes emerged, one of them related to gait problems in combination with balance problems and dizziness (LC2, 13.5%, gait-balance class), another class related to gait problems in combination with paralysis, weakness and spasms (LC4, 23.9%, gait-paralysis class), and a final class (LC3, 11.7%, fatigue-weakness class) that was characterized by weakness, fatigue, but also increased probabilities of dizziness, depression symptoms, memory, and gastro-intestinal problems. Differences between classes were also apparent in the average number of onset symptoms, which was about 9 in the multiple symptoms class, about 1.4 in the vision and paresthesia classes and about 4 in the other classes, but yielded no significant variation by sex or by MS type (results not shown).

When comparing the six class solution with the five and seven class solutions of the LCA, it turned out that the differentiation relates to gait problem classes. In the five class solution, there was only one gait problem class entailing both classes from the six class solution, gait-balance (LC2) and gait-paralysis (LC4), respectively. In the seven class model, the gait-paralysis class (LC4) divided in a class with less marked probabilities of gait-paralysis symptoms and a class with more pronounced and multiple symptoms, thereby incorporating also cases from the multiple symptoms class (LC1).

### MS-Specific Characteristics

The differences by sex between classes reflected the key symptoms [see sex ratio in gait problems and in the gait-paralysis class (LC4)]. The same applies to differences by clinical MS type (i.e., PPMS vs. other; see gait problems in LC4 and in PPMS; see details in Tables 1, 2). Here and in other instances (age at onset, number of relapses, EDSS proxy), the gait-paralysis class (LC4) took up one extreme, contrasted either by the fatigue-weakness class (LC3), or the vision problems (LC5) and the paresthesia class (LC6). The overall consequences as measured by the EQ5d visual analog scale were most burdening in the multiple symptoms class (LC1), whereas the psychological consequences as represented by the WHO-5 Well-Being Index were most serious in the fatigue-weakness class (LC3). In both instances, the paresthesia class (LC6) yielded the least burdening outcomes. Other MS-specific features, such as the use of immunomodulatory therapies and continuation of therapies, or familial aggregation, did not differ between classes.

**Table 2:** Latent classes based on MS onset symptoms: socio-demographic features, MS-specific variables and risk factors, frequencies with column % (except overall #)/proportions and means (SE); *p*-values related to χ^2^-test and to ANOVA, respectively.

	**LC1**	**LC2**	**LC3**	**LC4**	**LC5**	**LC6**	**Total**	***P*-value**
	**Multiple symptoms**	**Gait-balance**	**Fatigue-weakness**	**Gait-paralysis**	**Vision**	**Paresthesia**		
**Overall#** (row %)	273 (14.1)	262 (13.5)	228 (11.7)	465 (23.9)	414 (21.3)	300 (15.4)	1942 (100.0)	
**Socio-demographic features**								
**Sex**								0.007
Men	68 (24.9)	79 (30.2)	42 (18.4)	146 (31.4)	105 (25.4)	77 (25.7)	517 (26.6)	
Women	205 (75.1)	183 (69.8)	186 (81.6)	319 (68.6)	309 (74.6)	223 (74.3)	1,425 (73.4)	
**Age in 2019**	50.06 (0.76)	48.71 (0.87)	46.69 (0.81)	51.69 (0.59)	47.45 (0.61)	46.61 (0.72)	48.78 (0.29)	0.000
**Nationality**								0.677
Swiss	241 (88.3)	235 (89.7)	195 (85.5)	408 (87.7)	369 (89.1)	269 (89.7)	1717 (88.4)	
Other	32 (11.7)	27 (10.3)	33 (14.5)	57 (12.3)	45 (10.9)	31 (10.3)	225 (11.6)	
**Education**								0.007
Low	116 (57.1)	89 (47.6)	82 (48.8)	155 (47.8)	129 (41.6)	94 (41.4)	665 (46.9)	
High	87 (42.9)	99 (52.4)	86 (51.2)	169 (52.2)	181 (58.4)	133 (58.6)	754 (53.1)	
**MS-specific variables**								
**Ø symptoms**	8.81 (0.13)	4.30 (0.08)	4.90 (0.11)	3.25 (0.07)	1.57 (0.03)	1.41 (0.03)	3.72 (0.06)	0.000
**MS type**								0.000
PPMS	37 (13.6)	33 (12.6)	23 (10.1)	73 (15.7)	27 (6.5)	14 (4.7)	207 (10.7)	
CIS/RRMS	185 (67.8)	179 (68.3)	181 (79.4)	291 (62.6)	295 (71.3)	235 (78.3)	1366 (70.3)	
SPMS	51 (18.7)	50 (19.1)	24 (10.5)	101 (21.7)	92 (22.2)	51 (17.0)	369 (19.0)	
**Age of onset/Dx**	37.50 (0.70)	36.04 (0.73)	36.25 (0.64)	38.21 (0.52)	34.70 (0.50)	35.09 (0.57)	36.36 (0.24)	0.000
**Age of onset/1st Sx**	34.96 (0.70)	33.24 (0.68)	32.77 (0.62)	34.63 (0.51)	30.93 (0.46)	32.84 (0.56)	33.21 (0.23)	0.000
**#relapses (only RR / SP)**	6.66 (0.54)	5.68 (0.50)	6.04 (0.57)	7.29 (0.48)	5.77 (0.37)	5.01 (0.37)	6.10 (0.19)	0.010
**EDSS proxy**								0.000
1	138 (66.0)	129 (66.5)	131 (75.3)	208 (63.0)	236 (74.4)	190 (82.3)	1,032 (70.9)	
2	58 (27.8)	43 (22.2)	33 (19.0)	96 (29.1)	60 (18.9)	31 (13.4)	321 (22.1)	
3	13 (6.2)	22 (11.3)	10 (5.7)	26 (7.9)	21 (6.6)	10 (4.3)	102 (7.0)	
**IM therapies**								0.131
Yes	233 (86.3)	225 (87.5)	195 (85.9)	402 (87.0)	368 (89.8)	274 (92.3)	1,697 (88.2)	
No	37 (13.7)	32 (12.5)	32 (14.1)	60 (13.0)	42 (10.2)	23 (7.7)	226 (11.8)	
**No current IM therapy**								0.120
No current	54 (25.2)	47 (23.5)	38 (21.6)	102 (29.7)	75 (23.1)	47 (20.1)	363 (24.3)	
Current	160 (74.8)	153 (76.5)	138 (78.4)	242 (70.3)	250 (76.9)	187 (79.9)	1,130 (75.7)	
**Alternative medicine**								0.053
Yes	98 (42.6)	79 (35.7)	69 (34.3)	155 (38.2)	113 (31.6)	83 (31.3)	597 (35.5)	
No	132 (57.4)	142 (64.3)	132 (65.7)	251 (61.8)	245 (68.4)	182 (68.7)	1,084 (64.5)	
**EQ5d-VAS**	63.35 (1.41)	72.17 (1.39)	69.09 (1.43)	69.69 (1.02)	73.74 (1.07)	79.72 (1.16)	71.47 (0.50)	0.000
**WHO-5 Well-Being Index**	13.36 (0.34)	15.25 (0.35)	12.26 (0.41)	14.63 (0.27)	15.02 (0.28)	15.46 (0.35)	14.47 (0.13)	0.000
**Risk factors**								
**Familial aggregation**								0.289
No	214 (83.9)	196 (78.7)	163 (78.0)	341 (76.8)	319 (81.2)	222 (78.7)	1455 (79.4)	
Yes	41 (16.1)	53 (21.3)	46 (22.0)	103 (23.2)	74 (18.8)	60 (21.3)	377 (20.6)	
**Lifetime smoking**								0.002
Yes	139 (65.0)	103 (51.5)	111 (64.5)	199 (58.4)	171 (52.8)	116 (50.0)	839 (56.6)	
No	75 (35.0)	97 (48.5)	61 (35.5)	142 (41.6)	153 (47.2)	116 (50.0)	644 (43.4)	
**Alcohol consumption**								0.070
Never	31 (11.1)	22 (14.6)	29 (16.9)	46 (13.5)	20 (6.2)	28 (12.1)	176 (11.9)	
Less frequent than weekly	108 (50.7)	104 (52.3)	89 (51.7)	179 (52.5)	179 (55.2)	121 (52.4)	780 (52.7)	
Daily—weekly	74 (34.7)	73 (36.7)	54 (31.4)	116 (34.0)	125 (38.6)	82 (35.5)	524 (35.4)	
**BMI**	25.30 (0.34)	24.85 (0.36)	25.42 (0.44)	25.28 (0.30)	24.79 (0.28)	24.50 (0.28)	25.01 (0.13)	0.345

### Risk Factors and Comorbidities

The multiple symptoms class (LC1) stood out with regard to other potential risk factors. Notably, it was associated with a low education level (57.1 vs. 41–49% in other classes; see Table 2). Together with the fatigue-weakness class (LC3), it comprised a higher proportion of lifetime smokers (65%) than the other classes (50–58%).

These two classes repeatedly shared the highest proportions with respect to specific comorbid diseases and disorders (see Table 3): skin diseases, migraine, and—together with LC5 (vision problems)—cystitis, drug allergy, and angina/tonsillitis. Mononucleosis was most frequently reported in the fatigue-weakness class (LC3) and the paresthesia class (LC6) (~20 vs. 11–13% in other classes), whereas comorbidity with other autoimmune disorders appeared most frequently in the multiple symptoms class (LC1, 11.7 vs. 3–6% in other classes). Overall, the comorbidity patterns in the multiple symptoms and the fatigue-weakness class (LC3) were contrasted by low comorbidities in the gait-problem classes.

**Table 3:** Latent classes based on MS onset symptoms: lifetime comorbidities; frequencies with column % (positive answers); *p*-values related to χ^2^-test.

	**LC1**	**LC2**	**LC3**	**LC4**	**LC5**	**LC6**	**Total**	***p*-value**
	**Multiple symptoms**	**Gait-balance**	**Fatigue-weakness**	**Gait-paralysis**	**Vision**	**Paresthesia**		
Mononucleosis	23 (10.7)	26 (13.0)	33 (18.6)	40 (11.6)	44 (13.5)	49 (20.9)	215 (14.4)	0.007
Angina/Tonsillitis	74 (36.5)	55 (28.2)	73 (43.5)	89 (26.5)	115 (36.5)	82 (35.3)	488 (33.7)	0.001
Skin diseases	43 (20.1)	23 (11.5)	35 (19.9)	55 (16.0)	39 (12.0)	31 (13.7)	176 (15.1)	0.028
Herpes, fever blisters	14 (6.5)	17 (8.5)	19 (10.8)	31 (9.0)	18 (5.5)	17 (7.3)	116 (7.8)	0.313
Cystitis	28 (13.1)	22 (11.0)	26 (14.8)	30 (8.7)	33 (10.2)	20 (8.5)	159 (10.6)	0.219
Migraine	74 (34.6)	32 (16.0)	57 (32.4)	78 (22.7)	87 (26.8)	52 (22.2)	380 (25.5)	0.000
Gastro-intestinal disorders	19 (8.9)	21 (10.5)	18 (10.2)	19 (5.5)	21 (6.5)	16 (6.8)	114 (7.6)	0.186
Atopic diseases	78 (36.4)	67 (33.5)	69 (39.2)	127 (36.9)	119 (36.6)	83 (35.5)	543 (36.4)	0.850
Drug allergy	13 (6.1)	7 (3.5)	11 (6.3)	7 (2.0)	16 (4.9)	6 (2.6)	60 (4.0)	0.069
Other autoimmune disorders	25 (11.7)	11 (5.5)	8 (4.5)	11 (3.2)	14 (4.3)	8 (3.4)	77 (5.2)	0.000

### Multinomial Logistic Regression Analysis

Table 4 shows the results from the multinomial logistic regression analysis with the latent classes as the outcome variable. The fatigue-weakness class (LC3) was used as the reference category. The strong associations from the bivariate analyses were retained, whereas weaker associations relating to skin diseases and drug allergies were smoothed out. Forward and backward selection procedures yielded the same final model comprising sex, education level, and lifetime comorbidities with smoking, mononucleosis, angina/tonsillitis, migraine, and other autoimmune disorders as predictors.

**Table 4:** Multinomial logistic regression of latent classes on selected predictors, after backward selection procedure; category denoted by a dot is the reference category; *n* = 1,369.

		**LC1 vs. LC3**	**LC2 vs. LC3**	**LC4 vs. LC3**	**LC5 vs. LC3**	**LC6 vs. LC3**
	***P*-value (diff. of−2LLs)**	**Odds ratio (95% CI)**	**Odds ratio (95% CI)**	**Odds ratio (95% CI)**	**Odds ratio (95% CI)**	**Odds ratio (95% CI)**
Sex	0.043					
Men		0.64 (0.37–1.09)	0.50 (0.30–0.85)	0.53 (0.33–0.86)	0.59 (0.36–0.96)	0.81 (0.48–1.37)
Women		.	.	.	.	.
Education	0.020					
Low		1.53 (0.99–2.37)	1.15 (0.74–1.79)	1.12 (0.75–1.66)	0.85 (0.57–1.26)	0.83 (0.54–1.26)
High		.	.	.	.	.
Smoking	0.008					
Ever		0.92 (0.59–1.44)	0.56 (0.36–0.87)	0.72 (0.48–1.08)	0.60 (0.40–0.90)	0.53 (0.35–0.81)
Never		.	.	.	.	.
Mononucleosis	0.021					
Yes		0.50 (0.27–0.95)	0.90 (0.49–1.65)	0.79 (0.46–1.36)	0.68 (0.40–1.17)	1.32 (0.77–2.25)
No		.	.	.	.	.
Angina/tonsillitis	0.033					
Yes		0.88 (0.56–1.39)	0.62 (0.38–0.99)	0.54 (0.36–0.83)	0.83 (0.55–1.26)	0.72 (0.46–1.12)
No		.	.	.	.	.
Migraine	0.011					
Yes		1.01 (0.64–1.61)	0.45 (0.26–0.76)	0.78 (0.50–1.20)	0.88 (0.57–1.36)	0.64 (0.40–1.02)
No		.	.	.	.	.
Autoimmune disorders	0.007					
Yes		2.39 (1.02–5.60)	1.32 (0.50–3.49)	0.56 (0.20–1.53)	0.78 (0.30–1.99)	0.78 (0.28–2.14)
No		.	.	.	.	.

## Discussion

This study is among the first to explore the heterogeneity of MS through clustering of onset symptoms. It identified six typical configurations (classes) of onset symptoms that are characteristic for different groups of PwMS: a multiple symptoms class with many onset symptoms, three classes with four or five symptoms on average (gait-paralysis, gait-balance, fatigue-weakness), and two solitary classes (vision problems, paresthesia). Each symptom can belong to two or more classes and therefore can have fairly different implications. Similarly, MS characteristics (for example, the clinical MS subtype), comorbidities (for example, migraine, other autoimmune diseases) and potential risk factors (for example, upper respiratory tract infections, smoking) are differentially related to specific classes.

### Configurations of MS Onset Symptoms

The classes in this study represent typical configurations of MS onset symptoms. These configurations partly overlap with common theoretical assignments to dysfunction domains [e.g., motor, sensory, optic neuropathy, cerebellar/ataxia/brainstem (32)]; brainstem, sensory, bowel and bladder, cerebral, vision dysfunction in Tao et al. (33). This is underlined by the multiple symptoms class that was characterized by an overall increased symptom load. Other classes assembled fewer symptoms. With four to five symptoms on average, the fatigue-weakness class aggregated also dizziness and neuropsychiatric symptoms (depression symptoms, memory) which might be indicative of limbic pathway lesions in MS (34) and might shed a new light on the phenomenon of isolated cognitive relapses (35). Interestingly, bowel problems also featured in this class, suggesting that gastro-intestinal inflammation might have some effect (36). Gait problems can be mainly assigned to two separate classes with four to five onset symptoms that relate to pyramidal symptoms (paralysis) and cerebellar dysfunction (balance, dizziness). Finally, the analysis revealed two monosymptomatic classes: the vision problems and the paresthesia class. Research has already pointed at such solitary onset symptoms by labeling them as monofocal (37), monoregional (38), or single-attack (39).

The MS-related characteristics of the six classes comprised only slight dissimilarities between men and women, across the age of onset or between the general MS types (PPMS, RRMS, SPMS). In future, a better understanding of the classes will shed more light on these dissimilarities. Only marginal differences were found regarding familial aggregation and the use of immunomodulatory therapies.

### Details on Selected Risk Factors and Comorbidities

Table 5 summarizes the most important information on risk factors and comorbidities determined in this study: infectious mononucleosis and Epstein-Barr virus (EBV) infection, angina/tonsillitis, smoking, migraine, skin diseases, autoimmune diseases. Main and, if available, alternative interpretations of their associations with MS are added.

**Table 5:** Overview of risk factors related to MS onset symptom classes.

	**Involved latent classes**	**Findings**	**First line interpretation(s)**	**Alternative or specific interpretation(s)**
Infectious mononucleosis (IM)/Epstein Barr virus (EBV)	LC6 and LC3 with increased proportions LC4 and LC2 with decreased proportions	• IM is indicative of an EBV infection • EBV seropositive status is highly prevalent (>90% of population); hoever, persons with a seronegative status do not develop MS (40–42) • IM is more frequent when the EBV infection occurs later than in childhood, i.e., in adolescence and adulthood (43) • EBV the most securely established risk factor in MS (41, 44) • IM is *per se* an additional risk factor for MS (43)	• Higher proportions of IM hint at delayed EBV infections • A delayed EBV infection with (and without) IM increases the MS risk	• Subjects with a “resilient,” i.e., well-trained and well-regulated immune system less frequently experience manifest outcomes of common infections (30, 45), thus report also lower rates of mononucleosis (e.g., LC4 and LC2 members)
Angina/Tonsillitis	LC3 with increased proportion	• Tonsillitis is a risk factor of MS (46) • Tonsillectomy is a risk factor of MS (47–50)	• Upper respiratory tract inflammation (URTI) increases MS risk	• URTI (as a comprehensive category) does not predict RRMS (51) • The comorbidity of tonsillectomy with other autoimmune diseases [e.g., Crohn's disease (52, 53) and others (50)] indicates a more generalized deficiency of the immune system
Smoking	LC1 and LC3 with increased proportions	• Established risk factor of MS (54) • Also a risk factor regarding disease progression (55), including axonal desintegration (56) and a predictor of the number of functional domains involved (5) • Even passive smoking increases the risk of MS (57–59) • Snuff does not increase the risk of MS (60)	• Smoking contributes to URTI	• The comorbidity of smoking with many other autoimmune and chronical inflammatory diseases indicates a more generalized deficiency of the immune system
Migraine	LC3 with increased proportion	• Migraine increases the risk of MS and, vice versa, MS increases the risk of migraine (61)	• Migraine (in particular migraine with aura) could lead to an increase of the BBB permeability (61)	• Migraine could emerge in a pre-symptomatic MS phase (61)
Skin diseases	LC3 with increased proportion	• Reported associations between MS and skin diseases relate to psoriasis (62–64) • Onset of psoriasis preceding MS onset yields a severity-response relationship (63)	• Increased levels of TNF-α and IL17 in both diseases (63)	
Autoimmune diseases	LC1 with increased proportion	• Increased comorbidity with autoimmune diseases typically includes inflammatory bowel disease, thyroid disease, psoriasis (65–68) • Comorbidity between RA and MS may be reduced (69)	• The comorbidity with other autoimmune and chronical inflammatory diseases indicates a more generalized deficiency of the immune system	

### Characterizing the Latent Classes

In the following, we aim at providing a more precise picture of each latent class (LC). The multiple symptoms LC obviously assembles the worst features of MS risk: many different onset symptoms, different potential risk factors. Nevertheless, it is a peculiar LC. While MS is traditionally considered to be more frequent in middle- and upper socio-economic classes than in lower socio-economic classes (70–72), the multiple symptoms LC seems to be the exception; lower education level, less frequently reported infectious mononucleosis [signifying an earlier age of childhood infections (73) such as EBV], and increased smoking prevalence (74) are typical features of lower socio-economic classes. These findings are largely congruent with the predictors of a high number of impaired functional domains found in the study of Briggs et al. (5). The increased prevalence of other autoimmune diseases is marked in this class and indicates a more generalized deficiency of the immune system that goes beyond a selective predisposition for MS. Smoking enhances the probability of the association between MS and other autoimmune diseases (75).

The paresthesia and vision problems LCs show a contrasting picture. They are both associated with a higher educational level, a lower proportion of PPMS, fewer relapses than other LCs, and a lower age at onset. However, they differ intriguingly in the proportion of participants reporting mononucleosis. In comparison with the multiple symptoms class, it seems plausible that the pathogenic mechanisms in both monosymptomatic classes are limited or restricted in some way.

The fatigue-weakness LC was related to an increased rate of previous mononucleosis but also to conditions indicating upper respiratory tract inflammation (angina/tonsillitis and smoking) and possibly inflammation in the gut, suggested by bowel problems at onset and the trend association with drug allergy. The gait-paralysis LC has a less skewed sex ratio than other LCs, an older age of onset, a higher number of relapses and a higher proportion of PPMS. Despite the one-sided onset symptom profile, it shares these burdening features with the multiple symptoms LC. The profile of the gait-balance LC is similar but attenuated. In terms of comorbidities, both LCs with gait problems contrast with the fatigue-weakness and the multiple symptoms LC, with low proportions of angina/tonsillitis, drug allergy, and regarding LC2 also migraine and skin diseases.

### Strengths and Weaknesses of This Study

This study benefited from the large number of participants in the SMSR and the comprehensive assessment of different characteristics of MS. The methodology used in this study enabled a fine-grained analysis of MS subtypes. Typically, associations that are specific for subtypes vanish in analyses of overall data, or cause chronically inconsistent research findings.

The price to pay for an LCA in this context is the lack of comparability with healthy persons or controls—only internal comparisons are available at first attempt. The current analysis was confined to variables assessed with the initial and baseline questionnaires of the SMSR. Thus, some MS characteristics and several potential risk factors were not available for this analysis, notably vitamin D (76, 77), gastro-intestinal inflammation (36), traumatic experiences and stressful life periods (78). Moreover, no information about the onset year of any potential risk factor or comorbid condition was available.

For many PwMS, the beginning of pathological processes precedes onset symptoms for months or years. Thus, clinical onset symptoms might also include “later” symptoms and thus contain a certain amount of noisy information. Additional noise in the analyses emerged from the fact that we could not control for the age of onset of most conditions. We assume that they typically occur prior to the onset of MS.

This study shares further common limitations of studies based on self-reporting data. This includes various forms of recall bias and imprecise information contributing to more noise in the data.

Last but not least, classification models like the LCA come along with some specific problems. It is important to acknowledge that the potential number of classes in an LCA clearly depends on the sample size and the selection of variables introduced in the analysis. The model fit parameters rather help to identify the corresponding optimal range than to fix the exact number of classes. Therefore, the interpretability of the LCA outcomes plays also an important role.

A more specific concern relates to the fact that the LCA aims to group observations into homogeneous classes. Such a clear-cut delimitation of classes represents a rough simplification as is easily deducible from the multitude of onset symptom configurations in clinical practice. The simplification results in additional noise, which typically becomes apparent in subsequent analyses using the LCA outcomes.

## Conclusions

MS can be differentiated into several clusters along onset symptoms, thus revealing a new perspective on the heterogeneity of MS. These clusters comprise slight differences regarding MS characteristics such as clinical MS types (PP- RR-, SPMS), sex ratios, or age at onset, but they strongly diverge with regard to potential risk factors and to comorbidities. The clusters open prospects for a better understanding of basic issues in MS, such as relations between onset and later symptoms, differences between MS types, and, last but not least, the dynamics behind the current increase of MS incidence and prevalence figures.

## Data Availability Statement

The raw data supporting the conclusions of this article will be made available by the authors, without undue reservation.

## Ethics Statement

The studies involving human participants were reviewed and approved by Ethics Committee Zurich (Study number PB-2016-00894). The patients/participants provided their written informed consent to participate in this study.

## Author Contributions

VA-G designed and conducted the analysis and drafted the manuscript. NS assisted in the collection and maintenance of the data was involved in drafting of the manuscript. GH assisted in the collection and maintenance of the data and revised the manuscript for intellectual content. SR assisted in analysis of the data and revised the manuscript for intellectual content. MK assisted in the collection and maintenance of the data and revised the manuscript for intellectual content. YX assisted in interpretation of the analysis and revised the manuscript for intellectual content. CK assisted in the design of the study and revised the manuscript for intellectual content. JK assisted in the design of the study and revised the manuscript for intellectual content. Z-MM assisted in the design of the study and revised the manuscript for intellectual content. CZ assisted in the collection of the data and revised the manuscript for intellectual content. PC assisted in the design of the study and revised the manuscript for intellectual content. MP provided guidance on drafting of the manuscript and revised the manuscript for intellectual content. VW designed and conceptualized the study, provided guidance on interpretation of the data, and revised the manuscript for intellectual content. All authors contributed to the article and approved the submitted version.

## Conflict of Interest

The authors declare that the research was conducted in the absence of any commercial or financial relationships that could be construed as a potential conflict of interest.
