# Supplementary material for: Onset Symptom Clusters in Multiple Sclerosis: Characteristics, Comorbidities, and Risk Factors
Source: Front Neurol. 2021 Jul 6;12:693440. doi: 10.3389/fneur.2021.693440 (PMC8290323; doi:10.3389/fneur.2021.693440)
Supplement: Supplementary file 1 [file Data_Sheet_1.docx]

Table S1: Model fit indices derived from latent class analysis on onset symptoms, # of classes ranging from 1 to 7

Fit statistics 1-class 2-class 3-class 4-class 5-class 6-class 7-class

model model model model model model model

AIC 27147.7 25082.2 24672.6 24400.0 24234.4 24117.6 24069.4

BIC 27225.7 25243.8 24917.7 24728.7 24646.7 24613.5 24648.9

ABIC 27181.2 25151.6 24777.9 24541.2 24411.6 24330.7 24318.5

LMR-LRT, adj. 2077.2 435.7 299.9 193.8 145.5 77.5

p-value 0.000 0.000 0.000 0.027 0.006 0.356

bootstrap LRT 2095.5 439.6 302.6 195.5 146.7 78.1

p-value 0.000 0.000 0.000 0.000 0.000 0.000

Notes:

AIC = Akaike information criterion

BIC = Bayesian Information Criterion

ABIC = Sample-Size adjusted Bayesian Information Criterion

LMR-LRT adj. = Lo-Mendell-Rubin likelihood ratio test, adjusted

LRT = likelihood ratio test

Table S2: Model fit indices derived from latent class analysis on onset symptoms, men, # of classes ranging from 1 to 6

Fit statistics 1-class 2-class 3-class 4-class 5-class 6-class

model model model model model model

AIC 7086.9 6552.6 6446.0 6392.0 6357.8 6347.2

BIC 7146.3 6675.8 6632.9 6642.6 6672.2 6725.3

ABIC 7101.9 6583.8 6493.3 6455.3 6437.3 6442.8

LMR-LRT, adj. 558.2 135.1 83.1 63.4 40.2

p-value 0.000 0.001 0.005 0.107 0.201

bootstrap LRT 564.2 136.6 84.0 64.1 40.6

p-value 0.000 0.000 0.000 0.000 0.030

Notes:

AIC = Akaike information criterion

BIC = Bayesian Information Criterion

ABIC = Sample-Size adjusted Bayesian Information Criterion

LMR-LRT adj. = Lo-Mendell-Rubin likelihood ratio test, adjusted

LRT = likelihood ratio test

Table S3: Model fit indices derived from latent class analysis on onset symptoms, women, # of classes ranging from 1 to 7

Fit statistics 1-class 2-class 3-class 4-class 5-class 6-class 7-class

model model model model model model model

AIC 20055.1 18535.7 18247.5 18044.0 17915.7 17836.8 17804.6

BIC 20128.7 18688.3 18479.0 18354.5 18305.0 18305.1 18351.8

ABIC 20084.2 18596.2 18339.2 18167.1 18070.0 18022.4 18021.4

LMR-LRT, adj. 1535.2 315.3 231.3 156.9 107.8 61.7

p-value 0.000 0.001 0.000 0.036 0.214 0.046

bootstrap LRT 1549.3 318.2 233.4 158.3 108.8 62.3

p-value 0.000 0.000 0.000 0.000 0.000 0.000

Notes:

AIC = Akaike information criterion

BIC = Bayesian Information Criterion

ABIC = Sample-Size adjusted Bayesian Information Criterion

LMR-LRT adj. = Lo-Mendell-Rubin likelihood ratio test, adjusted

LRT = likelihood ratio test

Figure S1a-e:

Latent class analysis of onset symptoms in multiple sclerosis, men: probabilities of onset symptoms per class 1-5

Figure S2a-f:

Latent class analysis of onset symptoms in multiple sclerosis, women: probabilities of onset symptoms per class 1-6
